# Effects of different game-based physical activity programs on body composition in Chinese preschool children

**DOI:** 10.3389/fpubh.2025.1592084

**Published:** 2025-07-09

**Authors:** Sha Qu, Yueyu Feng, Wenjing Song, Zihao Wang, Zan Gao, Xing Zhao

**Affiliations:** ^1^School of Sport Science, Beijing Sport University, Beijing, China; ^2^School of Physical Education, Xihua University, Chengdu, China; ^3^Shanxi Sports Vocational School, Taiyuan, Shanxi, China; ^4^Department of Kinesiology, Recreation, and Sport Studies, The University of Tennessee, Knoxville, TN, United States

**Keywords:** body mass index, childhood obesity, sex differences, light physical activity, moderate-to-vigorous physical activity

## Abstract

**Background:**

Childhood obesity is a growing public health concern, with preschool obesity rates in China reaching 10.4%. Physical activity (PA) is crucial for childhood obesity prevention, yet many children fail to meet recommended PA levels. Existing research primarily examines BMI rather than a detailed analysis of body composition, with sex differences remaining uncertain. This study explores the effects of different 8-week PA interventions (LPA vs. MVPA) on body composition in Chinese preschoolers, offering valuable insights for early obesity prevention.

**Methods:**

This study utilized a two-arm experimental design with repeated measures, involving 86 preschool children from Beijing, China. Participants were randomly assigned to either the LPA or MVPA group and participated in an 8-week intervention, consisting of three 30-min sessions per week. Body composition was assessed pre- and post-intervention using bioelectrical impedance analysis (BIA), while dietary intake was evaluated through 24-h dietary recalls. Data analysis mainly included repeated-measures MANCOVA, with dietary intake controlled as a covariate.

**Results:**

There were no significant interaction effects of Group × Time, and Group × Time × Sex for any variables. A small but significant Sex × Time interaction for BMI was detected (*F* (1,71) = 4.41, *p* = 0.04, $\eta_{p}^{2}$ = 0.06). There was no significant group effect for any variable. Significant time effects were observed for total body water (TBW) (*p* < 0.01, $\eta_{p}^{2}$ = 0.17), skeletal muscle mass (SMM) (*p* < 0.01, $\eta_{p}^{2}$ = 0.18), body fat mass (BFM) (*p* < 0.01, $\eta_{p}^{2}$ = 0.11), percent body fat (PBF) (*p* = 0.04, $\eta_{p}^{2}$ = 0.06), fat-free mass (FFM) (*p* < 0.01, $\eta_{p}^{2}$ = 0.23), and FFM of Trunk (*p* = 0.05, $\eta_{p}^{2}$ = 0.05). Significant sex effects were observed. Boys demonstrated higher BMI, TBW, SMM, FFM, FFM of Right Arm, FFM of Left Arm, FFM of Trunk, FFM of Right Leg, and FFM of Left Leg than girls at both time points.

**Conclusion:**

PA showed a positive effect in preventing excessive BMI growth in boys, with MVPA potentially preventing the rapid accumulation of body fat. Compared to LPA, MVPA appears to yield greater benefits in increasing TBW, SMM, and FFM, while also being more effective in preventing rapid increases in BFM and PBF among preschool children. For girls, engaging in vigorous PA may be crucial for effectively enhancing body composition development.

## 1 Introduction

The World Health Organization (WHO) identifies childhood obesity as a critical global public health issue (1). The prevalence of childhood overweight and obesity is expected to rise by 60% worldwide, with projections indicating that approximately 250 million children will be affected by 2030 (2, 3). Recent data show that the prevalence of obesity among preschool children in China has sharply increased to 10.4% (4). This is particularly concerning as childhood obesity often continues into adulthood (5) and is strongly linked to a higher risk of premature mortality, diabetes, and cardiovascular disease later in life (6, 7). Additionally, Children who have not yet reached the body mass index (BMI) threshold for being overweight or obese may still be at a higher risk of developing obesity (1). Regular participation in physical activity (PA) is one of the most crucial interventions to combat childhood obesity and maintain a healthy body composition in children (8, 9). The WHO guidelines on physical activity, sedentary behavior, and sleep for children under 5 years of age recommend that children aged 3–4 years engage in at least 180 min of total physical activity (TPA) per day, including a minimum of 60 min of moderate-to-vigorous physical activity (MVPA) (10). The Exercise Guidelines for Children (3–6 years old), issued by the Chinese government in 2018, recommend that preschool-aged children participate in over 180 min of various PA daily, including at least 60 min of MVPA (11). However, many children still fail to meet the recommended amounts of TPA (12–15) and MVPA (16–19). Notably, only a small proportion of Chinese children and adolescents met the recommendations outlined in the Canadian 24-Hour Movement Guidelines (20, 21). For example, Yin et al. found that only 14.6% of Chinese preschoolers met the integrated guidelines for PA, sleep duration, and screen time (22). Furthermore, a comparative study across 11 countries from five major geographical regions revealed that Chinese children had the lowest average daily engagement in MVPA (23). Therefore, there is an urgent need to develop and implement effective and novel PA programs for Chinese preschoolers to prevent and curb childhood obesity.

Many studies have examined the impacts of PA on childhood overweight and obesity (24–26), and the finding among preschool children have been inconsistent (27, 28). Numerous studies have demonstrated the benefits of PA in preventing childhood overweight and obesity, showing that increased PA can help lower BMI or reduce obesity risk in preschool children (29–34). However, research findings yielded mixed results. Some studies suggested no significant differences in BMI between intervention and control groups after PA interventions (35–37). One study even suggested a positive association between PA and BMI-z scores, providing evidence that higher PA levels may even increase the likelihood of overweight or obesity in children (38). A systematic review examining the relationships between combinations of movement behaviors and health indicators reported that, the most ideal combinations of sedentary behavior and PA were both favorably and not associated with adiposity among toddlers and preschool-aged children (27). Similar studies conducted in China often adopt comprehensive policy-based intervention measures, primarily including dietary modifications, PA, health education, family involvement, and kindergarten-based initiatives (39–41). However, these studies typically consider PA, diet, health education, and other factors as an integrated whole, making it difficult to isolate and analyze the specific relationships between obesity and any single component of the multi-component intervention, such as the association between obesity and PA.

Notably, it is evident that research in this field has primarily relied on BMI and similar simple metrics, such as BMI-z scores, while more accurate and comprehensive measures of body composition have been less frequently utilized (42). Most studies investigating the associations between PA and body composition in preschool children used cross-sectional designs (43, 44). As a result, these studies can only identify correlations rather than confirm causation. A few longitudinal studies conducted in this area have produced inconsistent findings. In brief, Bürgi et al. and Metcalf et al. reported no association between PA and adiposity among preschool-aged children (45, 46). In contrast, Leppänen and associates identified a positive correlation between MVPA and vigorous VPA with the fat-free mass (FFM) index (47). The impact of PA intensity on preschool children’s body composition remains inconclusive. Fu and colleagues found that, as MVPA time increased in overweight preschool children, the reduction in percent body fat (PBF) occurred at a faster rate compared to light PA (LPA) (48). Conversely, Bürgi et al. found no longitudinal relationship between baseline MVPA and changes in body fat or BMI (45). Jimenez-Pavon suggested that while higher PA levels may contribute to obesity prevention, further research is needed, particularly in younger children (49). Thus, although an increasing number of studies (9, 48, 50) have begun to emphasize the importance of early childhood body composition development—rather than focusing solely on BMI and its derived indicators, as was the case in earlier research—findings regarding the effects of PA, particularly at different intensities, on body composition in preschool children remain inconsistent. In China, empirical research on the impact of PA interventions on body composition in preschool children remains unexplored, highlighting the urgent need for further studies to fill this literature gap. Furthermore, while existing literature has extensively documented the health benefits of MVPA, limited evidence exists regarding the impact of LPA on preschool children’s body composition. In a longitudinal study with 6,059 children, Agbaje et al. examined the effects of accelerometer-measured sedentary time and physical activity on DEXA- measured fat mass, which demonstrated that cumulative LPA was associated with decreased total fat mass and trunk fat mass from childhood through young adulthood. Although this association may be partially mediated by increased insulin levels, the pediatric population especially those with chronic diseases or mobility challenges could benefit from LPA (51). The researchers also emphasized that their findings require further validation across diverse ethnic populations. Additionally, further research is warranted to explore potential associations between LPA and other important body composition indicators in preschool-aged children.

Body composition, including the distribution of materials and tissues, varies significantly based on age and sex (52). Throughout most of childhood, boys and girls exhibit similar growth rates. However, on average, boys tend to have slightly greater height, body mass, BMI, total body water (TBW), skeletal muscle mass (SMM) and fat-free mass (FFM) compared to girls, whereas while girls generally have higher body fat mass (BFM) and percent body fat (PBF) (53–59). Studies conducted in China and other countries have also reported sex differences in PA (19, 60, 61). Societal norms and expectations often lead to boys receiving greater encouragement and more opportunities to engage in PA, contributing to higher PA levels among boys compared to girls (62). As a result, boys are more likely to engage in greater amounts of PA, particularly MVPA, in their daily lives (63–66). Chen et al., in their large-scale study, further confirmed that preschool boys accumulate significantly more daily MVPA than girls (67). Additionally, participate in higher levels of MVPA across various activity types on both weekdays and weekends than their female counterparts (68). Moreover, the impact of PA on body composition may also differ by sex (49, 69, 70). Previous studies have found associations between MVPA and body composition parameters, such as BMI and PBF, particularly in boys (49, 70). Specifically, Basterfield et al. reported a significant correlation between MVPA and fat mass index (FMI) as well as BMI z-scores in boys, while no such relationship was observed in girls (70). Jiménez-Pavón et al., in their systematic review, concluded that the link between MVPA and body composition variables was more consistent in boys than in girls. However, the underlying reasons for boys’ greater sensitivity to MVPA variations remain unclear (49). Ferrari and colleagues found that in boys, moderate PA (MPA), VPA, MVPA, and step counts were all associated with body composition variables, whereas in girls, only VPA showed a significant correlation, independent of sedentary behavior (69). Meanwhile, studies have shown that the PBF of girls was more influenced by LPA and sleep (71, 72). However, most existing studies are cross-sectional, limiting the ability to determine causal relationships. It remains unclear whether PA directly influences body composition or if it serves as an indicator of other lifestyle factors, such as dietary habits, that may contribute to body composition changes. Furthermore, sex differences in how PA affects body composition in Chinese preschool children remain largely unanswered. Therefore, further research is needed to examine these sex-specific associations in this population.

Additionally, most research has focused on the overall growth and development of body composition in children, with limited attention to regional balance. However, fat mass (FM) and FFM may be distributed unevenly across the body, such as between the arms and legs or between the limbs and trunk, influenced by factors like sex, age, and physical activity (73). Santarém et al. found that total and segmental FFM were linked to walking capacity, with upper and lower limb FFM in women and trunk FFM in men correlating with functional performance (74). Therefore, assessing both total and segmental FFM in early childhood is essential.

This study aims to explore how PA affects the body composition of Chinese preschool children. We created a curriculum for both LPA and MVPA, followed by an 8-week intervention. We hypothesized that, compared to LPA, MVPA has a more significant impact on body composition in preschool children and that the impact of PA on body composition varies by sex. This research aims to provide valuable insights that can inform the creation of early intervention strategies to combat overweight and obesity in children. By understanding the impact of PA on body composition, these findings will help shape targeted interventions in preschool children. Ultimately, the goal is to promote healthier lifestyles among preschool children in China, ensuring they develop habits that support long-term wellbeing.

## 2 Materials and methods

### 2.1 Research design and participants

This study employed a 2-arm experimental design with repeated measures. A total of 86 preschool children (50 girls; mean age: 5.62 ± 0.26 years) were recruited from a kindergarten in Beijing, China. The inclusion criteria were that children: (1) were 5–6 years of age, (2) had no cognitive, developmental, mental or motor impairments, and no fractures or other injuries, and (3) had parental consent and child verbal assent. The exclusion criterion was defined as attendance absences exceeding 20% of the total intervention sessions.

Cluster randomization was implemented at the classroom level to avoid contamination between intervention conditions, as the PA programs were delivered during scheduled physical education classes. The randomization procedure was conducted as follows: (1) Eligibility Screening: All four senior kindergarten classes met the inclusion criteria (e.g., similar class sizes, baseline body composition indicators). (2) Random Allocation: An independent statistician generated a randomization sequence using a computer-based random number generator (block randomization with a 1:1 ratio). Class identifiers (e.g., Class A, B, C, D) were concealed in opaque envelopes labeled with unique codes. After baseline assessments, the envelopes were opened sequentially by a research assistant uninvolved in recruitment or intervention delivery, revealing the group assignment (i.e., LPA or MVPA). (3) Implementation: Teachers and researchers were blinded to the allocation sequence until interventions began. Participants attended kindergarten for approximately 8 h per day, Monday through Friday. The intervention took place over an 8-week intervention period for 30 min per session (including 20 min of exercise intervention games and 10 min of warm-up and cool-down) 3 days per week. Children’s body composition indicators were measured before and after the intervention, while dietary intake was measured at baseline. This study was approved by the Sports Science Experiment Ethics Committee of Beijing Sport University.

### 2.2 Procedures

Participants were recruited from kindergarten classes with the help of classroom teachers. The teachers distributed flyers describing the study to parents when they picked up their children after school. Preschool children who returned signed consent forms were then screened by the researchers. Body composition was measured at both baseline and post-intervention using the same testing procedures. To minimize data loss, any child absent on a scheduled measurement day was assessed on an alternate day. A detailed body composition analysis report and exercise recommendations was given to each child’s parents as an incentive for successfully completing all data collection sessions.

### 2.3 Intervention conditions

Prior to the formal intervention, we pre-designed both LPA and MVPA sessions. The development of these sessions drew upon the guidelines provided in the “Guidelines for the Learning and Development of Children Aged 3–6” (75) and the “Exercise Guidelines for Children (3–6 years old)” (11). This approach ensured that the intervention content was aligned with the physical and psychological developmental characteristics of preschool children. PA games, which combine physical exercise with engaging activities, are an effective approach to promoting the growth and development of preschool-aged children (76, 77). Therefore, both the LPA and MVPA sessions in this study were designed in the form of PA games. Polar H10 heart rate monitors (Polar Electro Oy, Kempele, Finland) and ActiGraph GT3X + Link accelerometers (ActiGraph Corp., Pensacola, FL, USA) were used to validate the intensity of each intervention session. Any session in which more than 60% of the activity time met the prescribed intensity requirements for each group was considered to fulfill the criteria for formal intervention. The accelerometer sampling frequency was set at 100 Hz, with data aggregated into 15-s epochs. Intensity thresholds were defined using the Buttle cut-points (78): sedentary (0–60 counts/15 s), light (60–530 counts/15 s), moderate (530–1,112.5 counts/15 s), and vigorous (≥1,112.5 counts/15 s). A threshold of 60% of the maximum heart rate (HRmax) was used, with light intensity defined as < 60% HRmax and moderate-to-vigorous intensity defined as > 60% HRmax (79). The maximum heart rate was calculated as HRmax = 208–0.7 × age. All intensity validations of the sessions were conducted on children not included in the main study sample.

A total of 30 structured sessions (15 for LPA and 15 for MVPA) were developed, each comprising a 5-min warm-up (e.g., dynamic stretching and low-intensity games), a 20-min core phase, and a 5-min cool-down (guided breathing and stretching). While both groups shared identical frequency (3 sessions/week), duration (30 min/session), and 8-week timeline, the only variation in intensity between the two groups was achieved by modifying game density (i.e., number of games per session), movement complexity (e.g., walking versus running while carrying a basketball), and contextual roles (e.g., children acting as pandas [slow-paced] versus monkeys [rapid-paced] in games) to regulate the intensity of PA. All sessions underwent intensity validation before being included in the formal intervention to ensure compliance with research requirements.

The primary researcher worked closely with school administrators and teachers to integrate the intervention into the kindergarten curriculum. Intervention fidelity was continuously monitored across all components. PA interventionists underwent extensive training and were actively involved in session design, modification, and validation. All PA interventionists actively participated in the implementation of the formal 8-week intervention program throughout its duration. Weekly meetings between the lead researcher and interventionists ensured program consistency. The research team conducted weekly monitoring to ensure adherence to pre-established protocols. Regular assessments evaluated protocol compliance, content fidelity, activity intensity, and children’s engagement. Attendance for each session was documented by interventionists, and researchers subsequently compiled the records to calculate attendance rates. 87% of participants completed ≥19/24 sessions (e.g., LPA: 86%; MVPA: 88%).

### 2.4 Measures

#### 2.4.1 Body composition

Height and weight were measured according to the early childhood section of the China National Physical Fitness Determination Standard Manual, formulated by the State General Administration of Sports (80). Body mass index (BMI) was calculated from BMI = BM [kg] ÷ Height^2^ [m^2^].

In children, BMI assessment is usually interpreted using percentile charts. However, BMI alone does not offer detailed information about body fat mass (BFM). Overweight and obesity not only affect BFM and percent body fat (PBF) but can also lead to changes in other components of body composition. Dual-energy X-ray absorptiometry (DXA) is considered the” gold standard” for body composition assessment due to its safety, non-invasiveness, rapid scanning capability, and high precision (81). However, its widespread use among preschool-aged children is limited by the challenges of conducting on-site testing in kindergarten settings and the generally low acceptance rate among parents. Consequently, bioelectrical impedance analysis (BIA) has become one of the most widely used methods for estimating body composition, given its high accuracy, reproducibility, and suitability for field-based research outside laboratory settings (50, 82). Body composition was measured using an octapolar multifrequency bioelectrical impedance analyzer (Inbody models J30, Inbody, Seoul, Korea) at both baseline and post-intervention. The analysis allowed for the estimation of total body water (TBW), skeletal muscle mass (SMM), body fat mass (BFM), percent body fat (PBF), fat-free mass (FFM), free-fat mass of arm, leg and trunk. The tests were conducted after the children’s midday nap while standing, following the measurement guidelines (83).

#### 2.4.2 Dietary intake

Daily energy intake was estimated using validated 24-h dietary recalls (84), conducted on three weekdays and one weekend day. These recalls were performed at the beginning of the training program with assistance from parents and/or investigators. The dietary records were then analyzed using Boohee health software (Boohee Info Technology Co., Shanghai, China) to calculate energy intake. Participants were asked to maintain their current diet throughout the duration of the study.

### 2.5 Statistical analyses

Data were imported from Excel into an SPSS Version 29.0 (IBM Corp., Armonk, NY, USA) dataset for descriptive and inferential statistical analyses. Screening for outliers and non-normality was conducted before the main analysis. First, a descriptive analysis was conducted to describe the sample characteristics, including frequencies of sex and all variables’ means and standard deviations. Second, independent samples t-tests were used to determine if there were differences in body composition indicators between the LPA group and MVPA group at baseline. Last, a multivariate analysis of co-variance with repeated measures was used to examine changes in preschool children’s body composition indicators across time. Group (LPA group vs. MVPA group) and sex (boys vs. girls) were included as between-subject factors, time was treated as a within-subject factor, and dietary intake was included as a covariate. The significance level was set at 0.05 for all statistical analyses, with effect sizes reported for each comparison. Specifically, partial eta-squared ( $\eta_{p}^{2}$ ) was used as an index of effect size, for which small, medium, and large effect sizes were designated as 0.10, 0.25, and 0.40, respectively (85).

## 3 Results

*A priori* power analysis was performed using G*Power 3.1. Based on a repeated-measures MANCOVA design with two between-subject factors (Group and Sex), two time points (pre-test and post-test), and dietary intake as a covariate. With *α* = 0.05 and *β* = 0.20 (80% power), the analysis indicated a required sample of 64 participants (16 per Group × Sex cell) to detect significant Time × Group interactions. While sample size calculations were performed at the individual level, we recognize that the cluster randomization design (with classes as the unit of randomization) may have introduced intra-class correlations. This design effect could potentially reduce the effective sample size and statistical power.

To account for potential attrition, 86 children were recruited. A total of 11 participants were excluded from the analysis due to missing data or insufficient attendance rates. The final sample comprised 75 preschool children (44 girls; 5.60 ± 0.27 years, mean ± SD), which is deemed as appropriate to yield meaningful results. Detailed demographic characteristics and pre-test body composition indicators between LPA and MVPA group is displayed in Table 1.

**Table 1:** Demographic characteristics and pre-test body composition indicators between LPA and MVPA group.

Indicator	LPA group (*n* = 32)	MVPA group (*n* = 43)	*t*	*p*
Sex				
Girl	19 (59.37)	25 (58.14)		
Boy	13 (40.63)	18 (41.86)		
Age (year)	5.56 ± 0.27	5.64 ± 0.26	−1.26	0.21
Height (cm)	116.31 ± 4.69	116.56 ± 5.13	−0.22	0.83
Weight (kg)	20.19 ± 2.87	20.55 ± 3.05	−0.52	0.61
Dietary intake (kJ)	3,570.91 ± 298.15	3,483.38 ± 277.61	1.78	0.09
BMI (kg/m^2^)	14.85 ± 1.19	15.05 ± 1.31	−0.67	0.51
TBW (kg)	13.13 ± 1.65	13.10 ± 1.60	0.07	0.94
SMM (kg)	8.57 ± 1.35	8.54 ± 1.31	0.10	0.92
BFM (kg)	2.38 ± 1.07	2.76 ± 1.33	−1.34	0.18
PBF (%)	11.47 ± 4.10	12.99 ± 5.02	−1.39	0.17
FFM (kg)	17.82 ± 2.21	17.79 ± 2.18	0.05	0.96
FFM of right arm (kg)	0.68 ± 0.15	0.66 ± 0.14	0.49	0.63
FFM of left arm (kg)	0.67 ± 0.14	0.66 ± 0.14	0.28	0.78
FFM of trunk (kg)	7.32 ± 1.20	7.25 ± 1.10	0.27	0.79
FFM of right leg (kg)	2.03 ± 0.44	2.01 ± 0.42	0.25	0.81
FFM of left leg (kg)	2.03 ± 0.43	2.01 ± 0.41	0.20	0.85

Data are presented as number (%) or mean ± SD.

Pre-test data for children in the LPA group were comparable to those in the MVPA group with respect to age, height, weight, dietary intake, and body composition indicators (Table 1). Table 2 shows the descriptive results for the preschool children’s body composition indicators between intervention/sex groups and across time. Table 3 shows the results of the main effects and interaction effects analysis. Covariates appearing in the model are evaluated at the following values: Dietary intake = 3,533.57 (kJ).

**Table 2:** Descriptive statistics of children’s body composition indicators (mean ± SD).

Indicator	Pre-test			Post-test		
	Girl	Boy	All	Girl	Boy	All
LPA group (*n* = 32)						
BMI (kg/m^2^)	14.36 ± 0.83	15.58 ± 1.28	14.85 ± 1.19	14.37 ± 0.72	15.48 ± 1.29	14.82 ± 1.12
TBW (kg)	12.55 ± 1.15	13.98 ± 1.93	13.13 ± 1.65	12.73 ± 0.97	14.19 ± 1.75	13.33 ± 1.51
SMM (kg)	8.11 ± 0.93	9.25 ± 1.59	8.57 ± 1.35	8.26 ± 0.77	9.44 ± 1.44	8.74 ± 1.22
BFM (kg)	2.06 ± 0.83	2.83 ± 1.24	2.38 ± 1.07	2.34 ± 0.65	3.17 ± 1.54	2.68 ± 1.16
PBF (%)	10.64 ± 3.77	12.68 ± 4.41	11.47 ± 4.10	11.79 ± 2.73	13.67 ± 5.67	12.55 ± 4.20
FFM (kg)	17.05 ± 1.60	18.94 ± 2.56	17.82 ± 2.21	17.34 ± 1.34	19.28 ± 2.38	18.13 ± 2.04
FFM of right arm (kg)	0.61 ± 0.08	0.77 ± 0.18	0.68 ± 0.15	0.60 ± 0.07	0.76 ± 0.16	0.66 ± 0.14
FFM of left arm (kg)	0.61 ± 0.08	0.76 ± 0.17	0.67 ± 0.14	0.60 ± 0.07	0.74 ± 0.15	0.66 ± 0.13
FFM of trunk (kg)	6.86 ± 0.65	7.98 ± 1.51	7.32 ± 1.20	6.95 ± 0.57	8.12 ± 1.27	7.42 ± 1.07
FFM of right leg (kg)	1.88 ± 0.24	2.25 ± 0.57	2.03 ± 0.44	1.90 ± 0.20	2.26 ± 0.43	2.04 ± 0.36
FFM of left leg (kg)	1.88 ± 0.23	2.26 ± 0.54	2.03 ± 0.43	1.88 ± 0.19	2.27 ± 0.42	2.04 ± 0.35
MVPA group (*n* = 43)						
BMI (kg/m^2^)	14.87 ± 1.24	15.29 ± 1.39	15.05 ± 1.31	14.92 ± 1.10	14.98 ± 1.43	14.95 ± 1.24
TBW (kg)	12.63 ± 1.38	13.75 ± 1.70	13.10 ± 1.60	12.98 ± 1.39	13.85 ± 1.73	13.34 ± 1.58
SMM (kg)	8.18 ± 1.17	9.05 ± 1.36	8.54 ± 1.31	8.48 ± 1.12	9.13 ± 1.39	8.76 ± 1.26
BFM (kg)	2.71 ± 1.42	2.83 ± 1.25	2.76 ± 1.33	2.95 ± 1.41	2.84 ± 1.33	2.91 ± 1.36
PBF (%)	13.2 ± 5.58	12.69 ± 4.27	12.99 ± 5.02	13.88 ± 5.26	12.63 ± 4.46	13.35 ± 4.93
FFM (kg)	17.17 ± 1.89	18.66 ± 2.31	17.79 ± 2.18	17.65 ± 1.89	18.84 ± 2.33	18.15 ± 2.14
FFM of right arm (kg)	0.63 ± 0.14	0.70 ± 0.13	0.66 ± 0.14	0.64 ± 0.10	0.67 ± 0.13	0.65 ± 0.11
FFM of left arm (kg)	0.63 ± 0.14	0.70 ± 0.13	0.66 ± 0.14	0.64 ± 0.10	0.67 ± 0.13	0.65 ± 0.12
FFM of trunk (kg)	6.97 ± 1.03	7.63 ± 1.11	7.25 ± 1.10	7.24 ± 0.84	7.62 ± 1.15	7.40 ± 0.99
FFM of right leg (kg)	1.91 ± 0.42	2.15 ± 0.39	2.01 ± 0.42	1.98 ± 0.33	2.13 ± 0.43	2.04 ± 0.38
FFM of left leg (kg)	1.91 ± 0.41	2.15 ± 0.39	2.01 ± 0.41	1.98 ± 0.33	2.12 ± 0.43	2.04 ± 0.38

BMI, BM [kg] ÷ Height^2^ [m^2^]; TBW, total body water; SMM, skeletal muscle mass; BFM, body fat mass; PBF, percent body fat; FFM, free-fat mass.

**Table 3:** The results of the main effects and interaction effects analysis (F (p), $\eta_{p}^{2}$ ).

Indicator	Group	Time	Gender	Group × Time	Group × Gender	Gender × Time	Group × Time × Gender
BMI (kg/m^2^)	0.07 (0.797), 0.001	2.46 (0.121), 0.033	6.68 (0.012)*, 0.086	0.68 (0.412), 0.009	2.87 (0.095), 0.039	4.41 (0.039)*, 0.059	1.36 (0.248), 0.019
TBW (kg)	0.03 (0.864), 0.000	15.00 (<0.001)**, 0.174	12.25 (<0.001)**, 0.147	0.04 (0.839), 0.001	0.42 (0.522), 0.006	0.96 (0.332), 0.013	1.60 (0.211), 0.022
SMM (kg)	0.04 (0.848), 0.001	15.02 (<0.001)**, 0.175	11.53 (0.001)**, 0.140	0.07 (0.797), 0.001	0.50 (0.482), 0.007	1.09 (0.299), 0.015	1.76 (0.189), 0.024
BFM (kg)	0.67 (0.416), 0.009	8.73 (0.004)**, 0.109	1.98 (0.164), 0.027	1.46 (0.231), 0.020	1.94 (0.168), 0.027	0.29 (0.591), 0.004	0.96 (0.331), 0.013
PBF (%)	0.74 (0.394), 0.010	4.29 (0.042)*, 0.057	0.26 (0.609), 0.004	1.31 (0.256), 0.018	1.82 (0.181), 0.025	0.46 (0.499), 0.006	0.19 (0.666), 0.003
FFM (kg)	0.02 (0.878), 0.000	21.10 (<0.001)**, 0.229	11.82 (<0.001)**, 0.143	0.02 (0.903), 0.000	0.37 (0.544), 0.005	0.64 (0.427), 0.009	1.53 (0.220), 0.021
FFM of right arm (kg)	0.79 (0.376), 0.011	1.58 (0.213), 0.022	14.02 (<0.001)**, 0.165	0.01 (0.909), 0.000	3.71 (0.058), 0.050	1.45 (0.232), 0.020	0.66 (0.420), 0.009
FFM of left arm (kg)	0.32 (0.576), 0.004	1.54 (0.219), 0.021	12.26 (<0.001)**, 0.147	0.02 (0.879), 0.000	2.55 (0.115), 0.035	1.27 (0.264), 0.018	0.75 (0.390), 0.010
FFM of trunk (kg)	0.23 (0.632), 0.003	4.07 (0.047)*, 0.054	12.73 (<0.001)**, 0.152	0.04 (0.842), 0.001	1.80 (0.184), 0.025	0.92 (0.341), 0.013	1.83 (0.180), 0.025
FFM of right leg (kg)	0.14 (0.713), 0.002	0.39 (0.534), 0.005	10.85 (0.002)**, 0.133	0.09 (0.763), 0.001	1.00 (0.320), 0.014	0.62 (0.434), 0.009	0.43 (0.513), 0.006
FFM of left leg (kg)	0.12 (0.734), 0.002	0.28 (0.596), 0.004	11.81 (<0.001)**, 0.143	0.07 (0.796), 0.001	1.40 (0.241), 0.019	0.64 (0.428), 0.009	1.02 (0.315), 0.014

*p < 0.05, **p < 0.01.

There were no significant interaction effects of Group × Time for any variables. The Group × Sex interaction effect on FFM of the Right Arm was marginally significant (*F* (1,71) = 3.71, *p* = 0.06, $\eta_{p}^{2}$ = 0.05), suggesting a potential moderating role of sex in the observed effect. A small but significant Sex × Time interaction for BMI was detected (*F* (1,71) = 4.41, *p* = 0.04, $\eta_{p}^{2}$ = 0.06), while no significant interaction effects were found for other variables. Specifically, girls exhibited a significantly greater increase in BMI compared to boys, with a small effect size (Table 2). Moreover, there were no significant interaction effects of Group × Time × Sex for any variables (Table 3).

**Figure 1:**
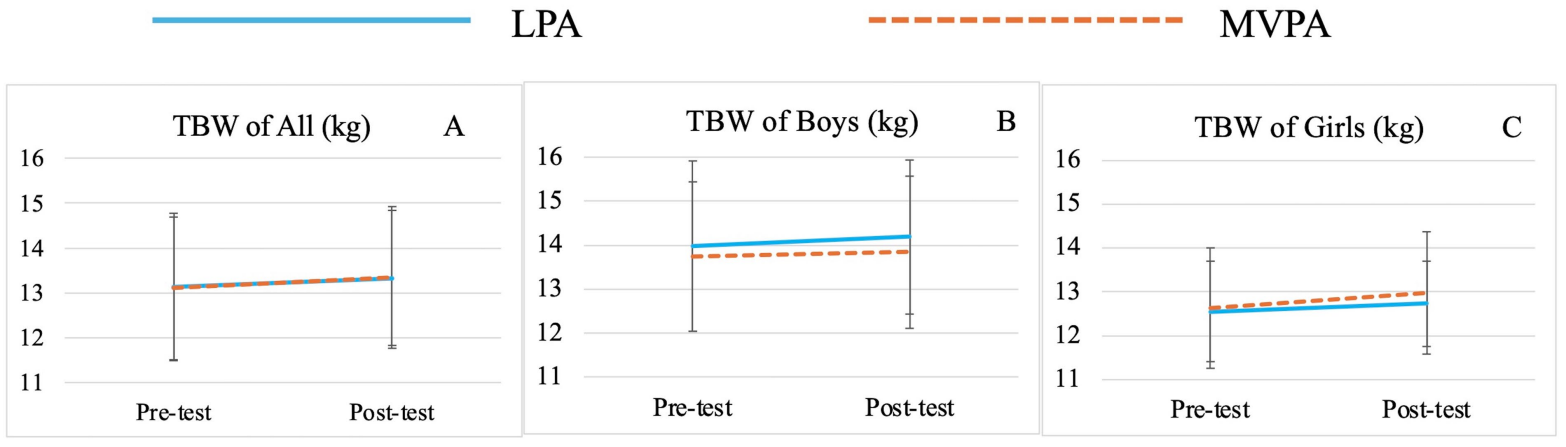
Changes of preschooler’s total body water (TBW) over time for the whole sample **(A)** and by genders (**B** for boys, **C** for girls).

**Figure 2:**
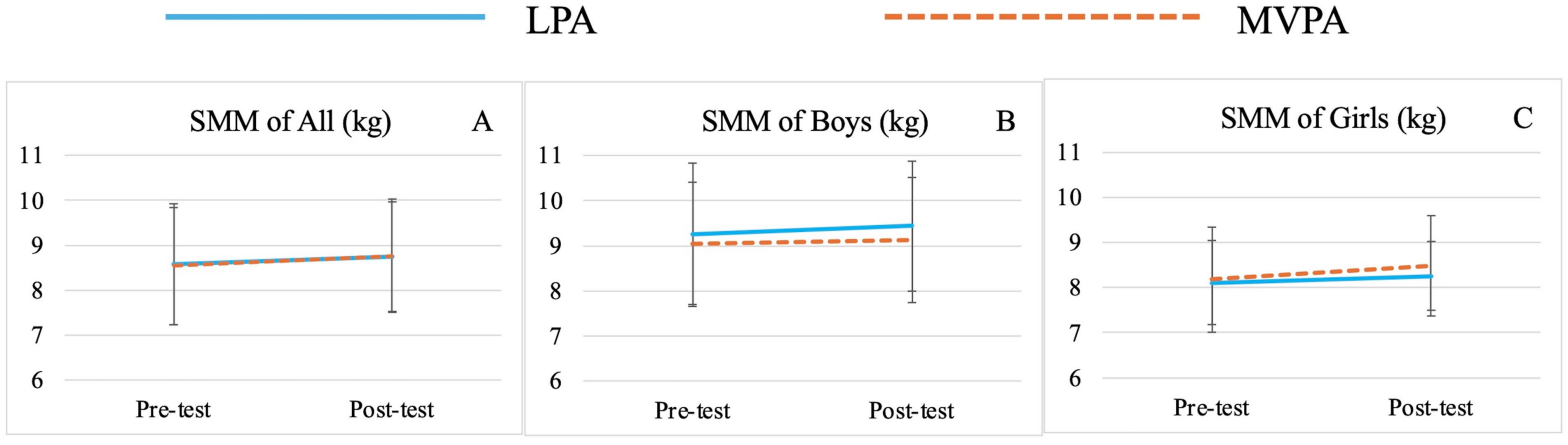
Changes of preschooler’s skeletal muscle mass (SMM) over time for the whole sample **(A)** and by genders (**B** for boys, **C** for girls).

**Figure 3:**
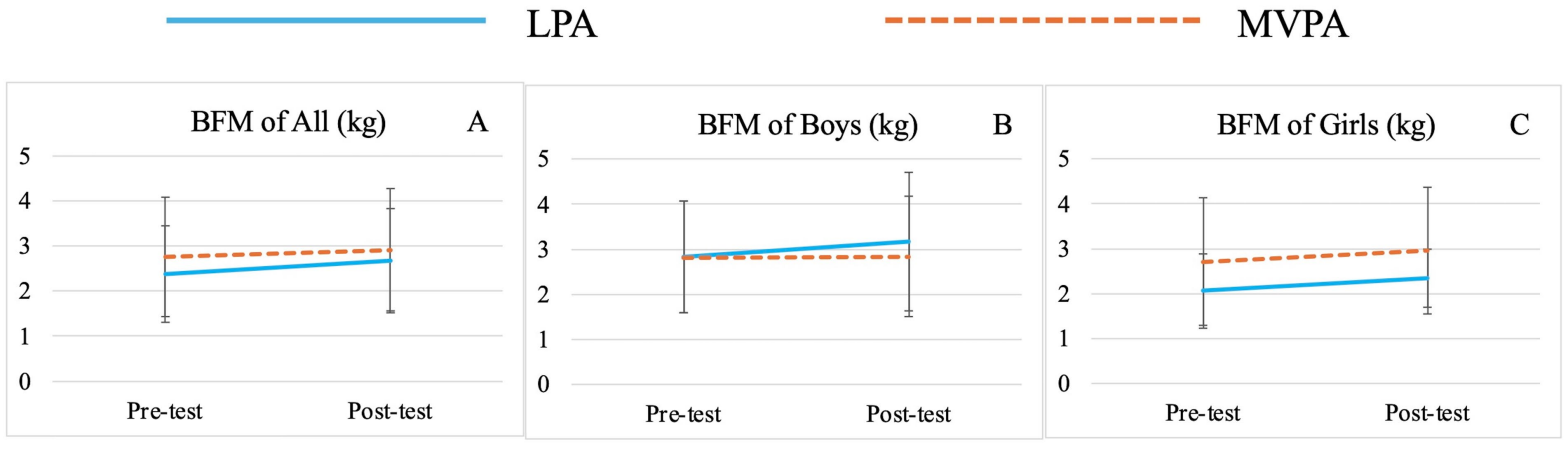
Changes of preschooler’s body fat mass (BFM) over time for the whole sample **(A)** and by genders (**B** for boys, **C** for girls).

**Figure 4:**
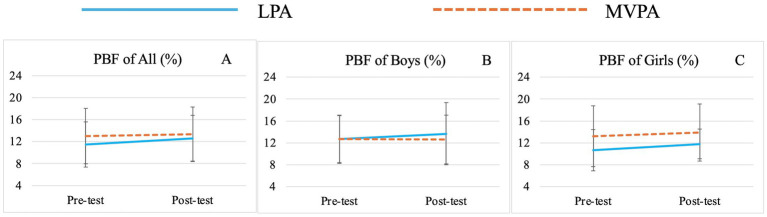
Changes of preschooler’s percent body fat (PBF) over time for the whole sample **(A)** and by genders (**B** for boys, **C** for girls).

**Figure 5:**
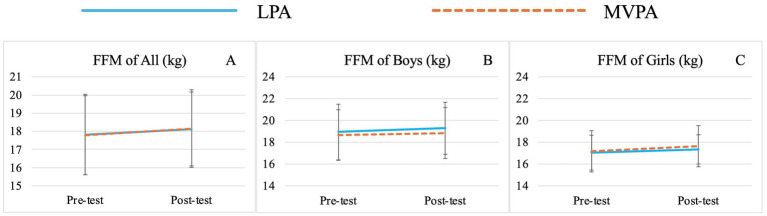
Changes of preschooler’s free-fat mass (FFM) over time for the whole sample **(A)** and by genders (**B** for boys, **C** for girls).

**Figure 6:**
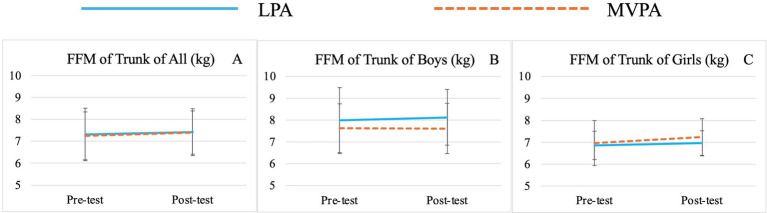
Changes of preschooler’s free-fat mass (FFM) of Trunk over time for the whole sample **(A)** and by genders (**B** for boys, **C** for girls).

**Figure 7:**
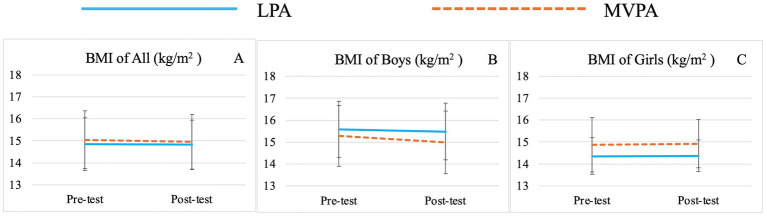
Changes of preschooler’s body mass index (BMI) over time for the whole sample **(A)** and by genders (**B** for boys, **C** for girls).

There was no significant group effect for any variable. Significant time effects were observed for TBW (*F* (1,71) = 15.00, *p* < 0.01, $\eta_{p}^{2}$ = 0.18) (Table 3; Figure 1A), SMM (*F* (1,71) = 15.02, *p* < 0.01, $\eta_{p}^{2}$ = 0.18) (Table 3; Figure 2A), BFM (*F* (1,71) = 8.73, *p* < 0.01, $\eta_{p}^{2}$ = 0.11) (Table 3; Figure 3A), PBF (*F* (1,71) = 4.29, *p* = 0.04, $\eta_{p}^{2}$ = 0.06) (Table 3; Figure 4A), FFM (*F* (1,71) = 21.10, *p* < 0.01, $\eta_{p}^{2}$ = 0.23) (Table 3; Figure 5A), and FFM of Trunk (*F* (1,71) = 4.07, *p* = 0.05, $\eta_{p}^{2}$ = 0.05) (Table 3; Figures 6A, 7A).

**Figure 8:**
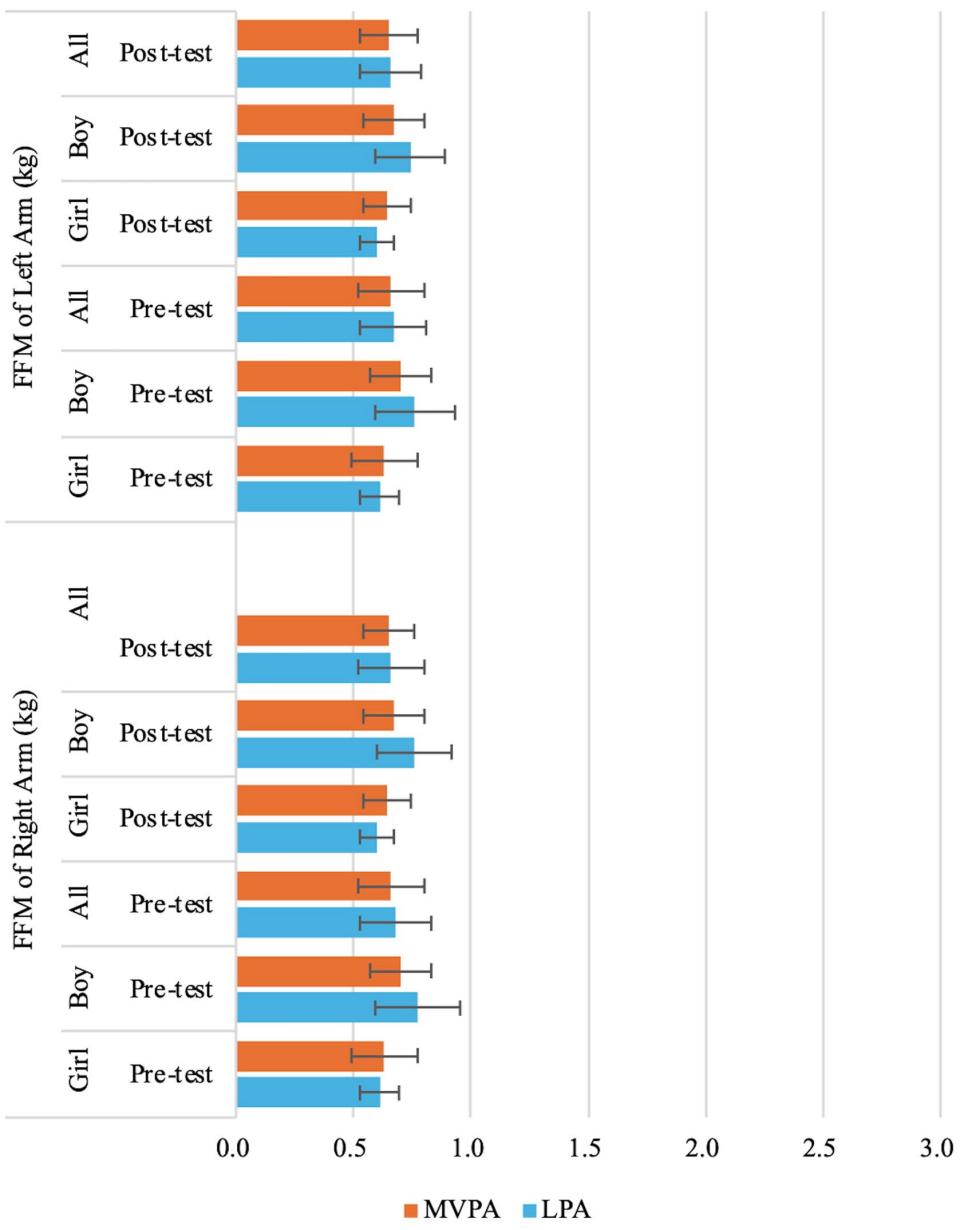
Changes of preschooler’s free-fat mass (FFM) of left arm and right arm over time for the whole sample and by genders.

**Figure 9:**
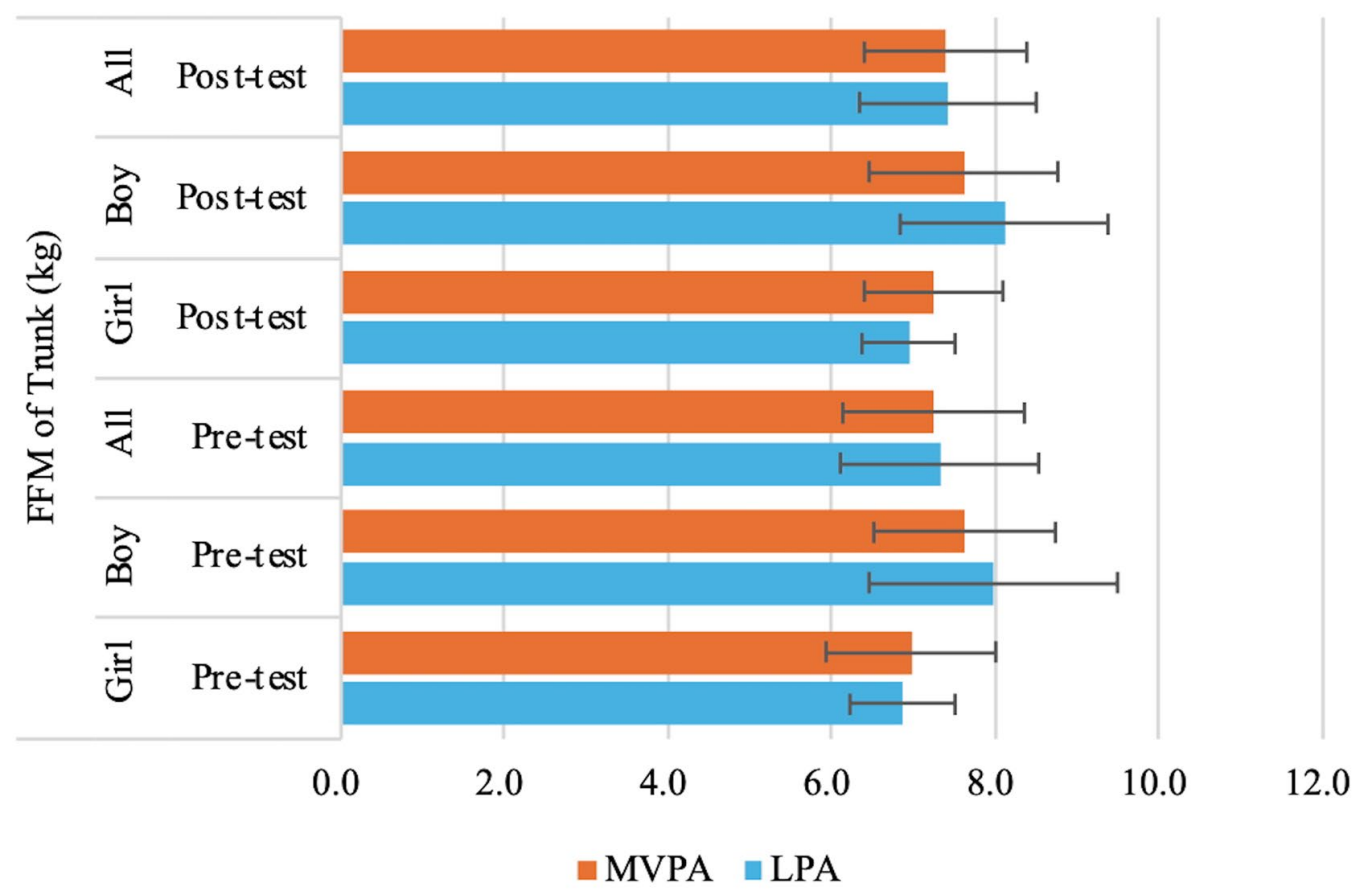
Changes of preschooler’s free-fat mass (FFM) of trunk over time for the whole sample and by genders.

**Figure 10:**
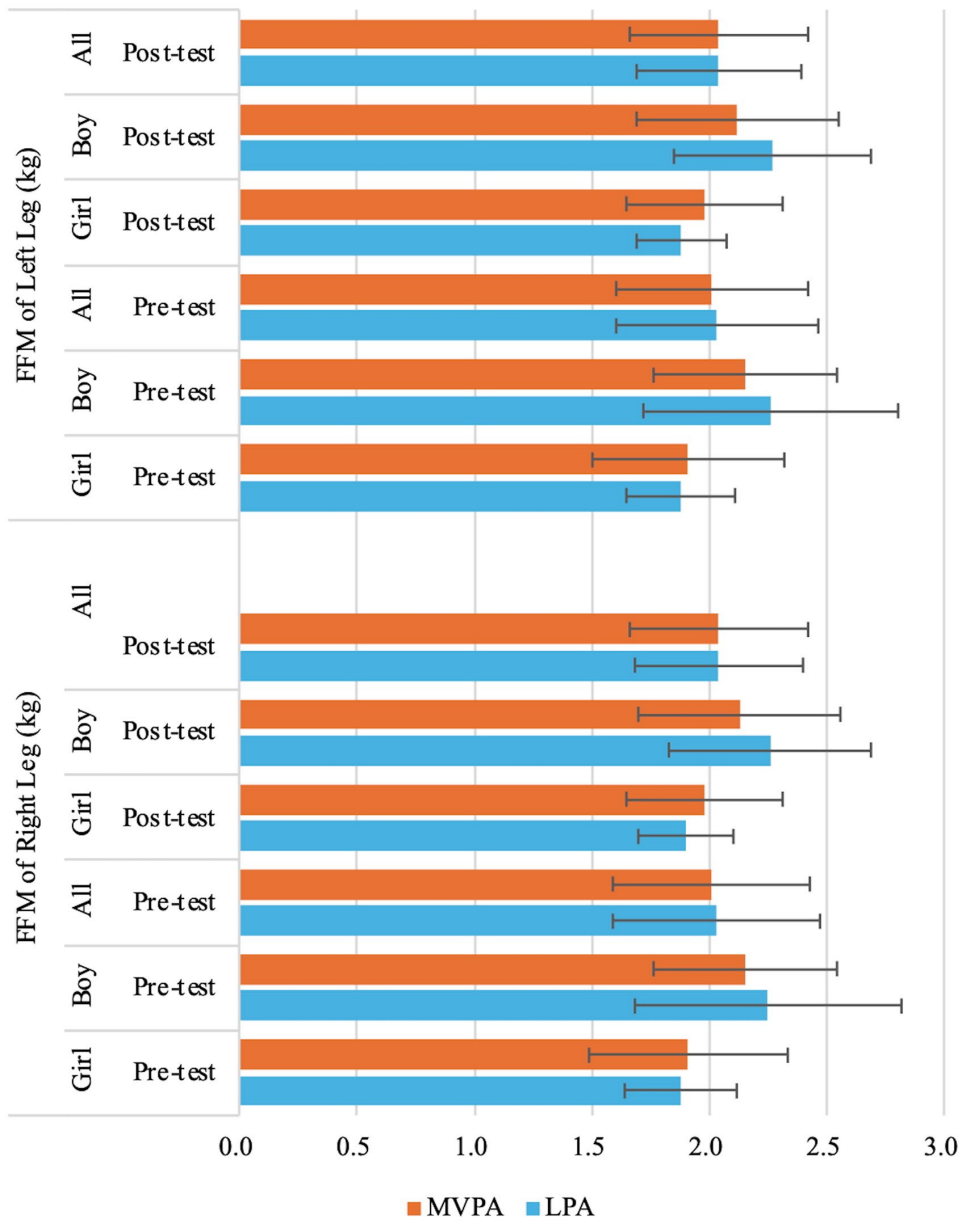
Changes of preschooler’s free-fat mass (FFM) of left leg and right leg over time for the whole sample and by genders.

Significant sex effects were observed for BMI (*F* (1,71) = 6.68, *p* = 0.01, $\eta_{p}^{2}$ = 0.09) (Table 3; Figures 7B,C), TBW (*F* (1,71) = 12.25, *p* < 0.01, $\eta_{p}^{2}$ = 0.15) (Table 3; Figures 1B,C), SMM (*F* (1,71) = 11.53, *p* < 0.01, $\eta_{p}^{2}$ = 0.14) (Table 3; Figures 2B,C), FFM (*F* (1,71) = 11.82, *p* < 0.01, $\eta_{p}^{2}$ = 0.14) (Table 3; Figures 5B,C), FFM of Right Arm (*F* (1,71) = 14.02, *p* < 0.01, $\eta_{p}^{2}$ = 0.17) (Table 3; Figure 8), FFM of Left Arm (*F* (1,71) = 12.26, *p* < 0.01, $\eta_{p}^{2}\;$ = 0.15) (Table 3; Figure 8), FFM of Trunk (*F* (1,71) = 12.73, *p* < 0.01, $\eta_{p}^{2}$ = 0.15) (Table 3; Figures 6B,C, 9), FFM of Right Leg (*F* (1,71) = 10.85, *p* < 0.01, $\eta_{p}^{2}$ = 0.13) (Table 3; Figure 10), and FFM of Left Leg (*F* (1,71) = 11.81, *p* < 0.01, $\eta_{p}^{2}$ = 0.14) (Table 3; Figure 10). Specifically, boys demonstrated higher BMI, TBW, SMM, FFM, FFM of Right Arm, FFM of Left Arm, FFM of Trunk, FFM of Right Leg, and FFM of Left Leg than girls at both time points (Table 2).

## 4 Discussion

The healthy development of body composition during early childhood is vital for subsequent growth and development (86). PA games, which blend physical exercise with playful activities, are particularly important for the growth of preschool-aged children (76, 77). In this study, both types of PA interventions were delivered through PA games. Individual-level randomization was deemed impractical because the intervention required modifying structured physical education sessions at the classroom level. Cluster randomization minimized logistical conflicts (e.g., differing activity protocols within the same class) and ensured fidelity to the intervention design. This study assesses the effects of a school-based intervention on the body composition of Chinese preschool children by examining changes in body composition indicators before and after an 8-week PA game intervention, conducted at equal doses but differing in intensity (LPA vs. MVPA).

In this study, we found no significant interaction effects of Group × Time for body composition indicators. Our finding of no significant change at the 8-week follow-up is consistent with previous obesity prevention studies in both younger (87) and older children (88, 89). This may primarily be attributed to the fact that the majority of preschool children included in this study were within the healthy weight range. Yavuz et al. found that interventions focusing solely on overweight and obese children were more effective than those including children with a healthy weight (90). Had this study exclusively targeted overweight and obese children, the intervention effects might have been more pronounced. However, this study intentionally included healthy-weight children, emphasizing the importance of prevention in combating childhood obesity. Encouraging the early adoption of healthy behaviors and supporting long-term weight maintenance is essential for all children (91). Additionally, it is plausible that the 8-week intervention might be too short to yield positive effects on most study variables. Thus, a longer intervention period may be necessary to detect significant differences in body composition changes between groups.

Among the remaining interaction effects, this study only identified a significant Sex × Time interaction. It is observed that the changes in BMI differed between boys and girls over the 8-week PA intervention. In detail, girls had significantly greater increase in BMI than those in boys. During childhood, developmental trajectories of growth are largely comparable between sexes. Nevertheless, sex-specific differences in body composition emerge, with boys demonstrating moderately higher values in anthropometric indices (height, body mass, BMI) (53–59). The findings indicate that, compared to girls, boys’ BMI is more sensitive to PA. From the perspective of growth and development, research suggests that BMI in preschool-aged children typically displays a pattern of initial increase, followed by a decline—most notably between ages 4 and 5—before gradually rising again (104). In this study, the preschool children had a mean age of 5.6 years at baseline, a stage when BMI is expected to begin increasing. This further illustrates that, compared to girls, the PA intervention was more effective in moderating excessive BMI increases among boys.

Similarly, no significant group effects on body composition indicators were observed in this study. As previously mentioned, research on obesity or body composition interventions targeting preschool children remains limited (36), and the findings of existing studies have been mixed (27, 28). In brief, Waters and colleagues in their review identified only eight obesity prevention intervention studies focused on children under 5 years old. When examining the combined results, researchers found that BMI change from pre- to post-intervention was 0.26 units lower in the intervention group compared to the control group, though this difference was not statistically significant (92). Another systematic review of obesity intervention programs targeting Hispanic preschool children included eight studies, all incorporating nutrition and PA components, with some also addressing parenting guidance and social networking. However, only four of these studies reported significant reductions in children’s weight, BMI, or BMI-z scores (93). Overall, obesity prevention interventions in preschool children have shown differential effects on weight change and adiposity, highlighting the challenges of addressing obesity in this population (36). Interestingly, studies incorporating multiple intervention components have demonstrated limited effectiveness in improving BMI and body composition outcomes. Although the intervention was implemented rigorously in the present study, no significant main group effect on body composition was observed. However, this study still adds valuable insights to the evidence base for childhood obesity prevention. Previous research suggests that interventions targeting one or two specific behaviors (e.g., PA, diet) tend to yield the most effective outcomes (94, 95). This rationale underscores our study’s approach of controlling for dietary intake variables and focusing solely on changes in PA.

Although group effects on various body composition indicators were not observed in this study, preschoolers’ TBW, SMM, BFM, PBF, FFM and FFM of Trunk significantly improved from baseline to post-intervention regardless of group and sex affiliations. It should be acknowledged that this may be attributed to the natural growth and development of young children. However, greater increases in TBW, SMM, FFM and FFM of Trunk were still observed in the MVPA group over time, while greater increases in BFM and PBF were noted in the LPA group. The lack of statistical significance may be attributed to the relatively short intervention duration of 8 weeks and relatively small sample size. Nevertheless, these findings still provide valuable insights, to some extent, MVPA offers superior advantages in promoting the growth of TBW, SMM and FFM, while also being more effective in preventing the rapid increase in BFM and PBF in preschool children. This observation is consistent with those of previous studies (47, 69, 96–100). For example, Leppänen et al. found that higher levels of vigorous PA (VPA) and moderate-to-vigorous PA (MVPA) at age 4.5 were significantly linked to greater fat-free mass (FFM) after 12 months, suggesting that promoting high-intensity PA at an early age may have lasting benefits for body composition (47). Wyszyńska and colleagues, in their cross-sectional study with preschoolers, reported that boys engaging in less than 60 min of MVPA per day had twice the risk of obesity compared to peers who met the 60-min threshold (101). Similar trends have been observed in school-aged children and adolescents. A study on Brazilian children found that MVPA and daily step counts, particularly VPA, were associated with body composition independent of sedentary behavior (69). Research on adolescents further supports these findings, showing that VPA more effectively reduces subcutaneous fat and increases muscle mass (96–99). Adolescents engaging in VPA tend to have better body composition indicators, reflecting a higher proportion of metabolically active lean mass (102).

Significant sex effects were observed for BMI, TBW, SMM and FFM, with boys consistently showing higher values across time, aligning with previous research (53–59). Studies indicate that sex differences in body composition emerge early, with boys exhibiting greater BMI (53, 56, 59), TBW (58, 59), SMM (58, 59), FFM (53, 54, 59) than girls. However, prior research has also found that while boys generally have higher body composition indicators, girls tend to have greater BFM (53, 58, 59) and PBF (53–55, 57–59, 103). This study did not find significant sex differences in BFM or PBF. Within the MVPA group, boys initially had higher BFM than girls, despite having lower PBF, suggesting that their greater body weight contributed to more fat mass without an elevated body fat percentage. Notably, after the intervention, boys’ BFM decreased below that of girls, reinforcing the idea that MVPA may be particularly effective in reducing excess fat accumulation and controlling BMI growth in boys. Previous studies have highlighted sex differences in how PA impacts body composition. Among boys, MVPA was significantly associated with reductions in body fat indicators, while for girls, only VPA showed similar effects, independent of sedentary behavior (69). These findings suggest that for girls, VPA may be necessary to achieve meaningful improvements in body composition.

In addition to analyzing the overall changes in whole-body composition, this study also examined changes in FFM across specific body regions, an approach rarely seen in previous research (73, 74). The findings indicated significant sex effects in FFM of Right Arm, FFM of Left Arm, FFM of Trunk, FFM of Right Leg, and FFM of Left Leg, with boys exhibiting higher values for these indicators than girls across time. Moreover, differences in FFM between boys and girls were most pronounced in the trunks, followed by the legs, and least noticeable in the arms. After the intervention, although overall fat-free mass (FFM) increased in both boys and girls, FFM in the right and left arms decreased post-intervention, particularly among boys and girls in the LPA group and boys in the MVPA group. This suggests that body composition development in early childhood may not occur uniformly across different body regions. The results may be influenced by the nature of the PA programs, which primarily focused on running and jumping games, leading to limited upper limb engagement. This highlights an important consideration: designing PA programs for young children should incorporate exercises that target all body regions to support balanced body composition development.

This study had the following strengths: (1) It was one of the few known studies to integrate same dose but two different intensity of PA games into the school curriculum for preschool-aged children; (2) It considered the impact of food intake on body composition. After baseline dietary intake assessments, participants were instructed to maintain their usual dietary habits throughout the intervention period, minimizing potential confounding effects from dietary intake; and (3) It included a comprehensive range of objective body composition indicators, such as TBW, SMM, BFM, PBF, FFM, FFM of the arm, leg, and trunk. By examining the effects of different PA programs on these indicators, the study provides valuable and up-to-date information for parents, teachers, and researchers about the impact of PA on overall body composition and the composition of different body regions in young children. It provides theoretical support for enhancing awareness among parents and professionals in the field about early childhood growth and development, highlighting the critical importance of early attention to body composition development.

However, this study has several limitations. First, an important limitation is that our study included only four classes, which may have constrained our ability to detect small effect sizes. The cluster randomization design, while appropriate for our intervention, means that the effective sample size may be smaller than the nominal number of individual participants due to potential intra-class correlations. Future studies with larger numbers of clusters would help address this limitation. Second, while the study employed a quasi-experimental design, the absence of a true no-intervention control group precludes definitive causal inferences regarding intervention effects versus natural developmental changes. Third, despite the rigorous training for the researchers involved, the intervention duration was relatively short. Longer intervention periods are recommended for future studies. Fourth, due to class schedule constraints, it was not possible to randomly assign children to different classrooms, which resulted in the absence of individual-level randomization. Future studies should implement cluster randomized controlled trials across multiple kindergartens to minimize threats to the internal validity of the experiment. Additionally, the PA programs implemented in this study primarily focused on locomotor skills (e.g., running, and jumping), which may have resulted in limited upper limb engagement. Future research should incorporate upper-body-dominant activities (e.g., ladder climbing, and beanbag throwing) to investigate the region-specific effects of PA interventions on body composition. Lastly, while our current analysis accounted for clustering through appropriate methods, we acknowledge that employing hierarchical linear modeling (HLM) or generalized estimating equations (GEE) could provide additional robustness by more fully accounting for intra-cluster correlations. Future studies incorporating these advanced analytical approaches may yield further insights into these relationships.

## 5 Conclusion

The findings of this study provide new insights into the impact of PA programs of same dose but varying intensities on body composition in Chinese preschool children. PA showed a positive effect in preventing excessive BMI growth in boys, with MVPA potentially preventing the rapid accumulation of body fat. Compared to LPA, MVPA appears to have greater benefits in increasing TBW, SMM, and FFM, while also being more effective in preventing rapid increases in BFM and PBF among preschool-aged children. For girls, engaging in vigorous PA may be crucial for effectively enhancing body composition development. Future studies should identify different segments on children’s PA behaviors both at school and outside of school (105). High-quality intervention studies incorporating health education control groups and extended durations (e.g., ≥12 weeks) are needed to explore the role of PA at varying intensities in improving body composition and other health indicators among preschool children, and to systematically investigate the mechanisms underlying sex-specific responses to PA.

## Data Availability

The original contributions presented in the study are included in the article/supplementary material, further inquiries can be directed to the corresponding authors.
